# Pilot randomized trial demonstrating reversal of obesity-related abnormalities in reward system responsivity to food cues with a behavioral intervention

**DOI:** 10.1038/nutd.2014.26

**Published:** 2014-09-01

**Authors:** T Deckersbach, S K Das, L E Urban, T Salinardi, P Batra, A M Rodman, A R Arulpragasam, D D Dougherty, S B Roberts

**Affiliations:** 1 Division of Neurotherapeutics, Department of Psychiatry, Harvard Medical School, Boston, MA, USA; 2 Energy Metabolism and Obesity Laboratory, Jean Mayer USDA Human Nutrition Research Center on Aging, Tufts University, Boston, MA, USA

## Abstract

**Objectives:**

Obesity is associated with hyperactivation of the reward system for high-calorie (HC) versus low-calorie (LC) food cues, which encourages unhealthy food selection and overeating. However, the extent to which this hyperactivation can be reversed is uncertain, and to date there has been no demonstration of changes by behavioral intervention.

**Subjects and methods:**

We used functional magnetic resonance imaging to measure changes in activation of the striatum for food images at baseline and 6 months in a pilot study of 13 overweight or obese adults randomized to a control group or a novel weight-loss intervention.

**Results:**

Compared to controls, intervention participants achieved significant weight loss (−6.3±1.0 kg versus +2.1±1.1 kg, *P*<0.001) and had increased activation for LC food images with a composition consistent with that recommended in the behavioral intervention at 6 months versus baseline in the right ventral putamen (*P*=0.04), decreased activation for HC images of typically consumed foods in the left dorsal putamen (*P*=0.01). There was also a large significant shift in relative activation favoring LC versus HC foods in both regions (*P*<0.04).

**Conclusions:**

This study provides the first demonstration of a positive shift in activation of the reward system toward healthy versus unhealthy food cues in a behavioral intervention, suggesting new avenues to enhance behavioral treatments of obesity.

## Introduction

Mechanisms that ensure sufficient food consumption for bodily maintenance and health are integral to the survival of all species including humans. As the gathering and preparation of food requires work, neurological pathways that provide reward for the anticipation of eating are essential for incentivizing the necessary effort.^1^ Abnormalities in the anticipatory reward system are implicated in the development of obesity and the frequent resistance of obesity to successful treatment.^2,3^ In particular, a conditioned hyperactivation of the reward system for high-calorie (HC) versus low-calorie (LC) food cues may be of particular importance because HC foods are readily available and easily overeaten, known to provide more reward than LC foods,^4,5^ and an individual's relative reward center activation to HC versus LC cues will influence which foods are selected for consumption when a wide array of foods is available. However, to our knowledge, the only reported assessments of changes in reward system responsiveness to HC versus LC foods during behavioral weight-loss programs have reported no significant changes.^6,7^ Thus, whether neural plasticity can be achieved to reverse reward system hyperactivation for HC versus LC foods to facilitate obesity treatment is unknown.

We recently reported a first demonstration of reduced hunger combined with reduced food cravings in a long-term behavioral intervention involving consumption of regular food.^8, 9, 10^ Here, we report a pilot study examining changes in anticipatory reward circuitry activation to different type of foods in individuals randomized to the same behavioral intervention or to a wait-listed control.

## Subjects and methods

### Participants

Subjects completing this 6-month pilot study were 13 healthy adult overweight or obese men and women who were part of a randomized trial of a novel weight-loss intervention in worksites.^8^ Inclusion criteria for the main trial included being employed by one of the four worksites that were participating in the trial, being ⩾21 years old, having a body mass index ⩾25 kg m^−2^ and providing a doctor's note supporting enrollment in a weight-loss program. Additional inclusion criteria for this functional magnetic resonance imaging (fMRI) study included being <65 years old, right handed and having normal or corrected-to-normal vision and hearing. Exclusions included any current or past (within 2 years) psychiatric history including use of medications prescribed for psychiatric diagnoses other than depression, or any MRI contradiction including a metal implant or history of claustrophobia. Thirteen out of 15 individuals who enrolled in the study (8 intervention, 5 controls) completed both the main protocol and fMRI scans for this study. The two subjects who did not complete had emerging exclusions (one was laid off from her job during the study so could not participate in the main study, and one experienced claustrophobia during his baseline MRI scan). Subject characteristics of the completers are shown in Table 1. The study was approved by the Tufts Medical Center Institutional Review Board and the Massachusetts General Hospital Institutional Review Board, and all subjects provided written informed consent prior to enrollment.

### Control and intervention groups

Following baseline outcome assessments that included measures of weight and eating behavior variables as described elsewhere,^10, 11, 12^ the worksites were randomized to control and intervention treatments. Control subjects were wait-listed to receive a weight control intervention starting in 6 months and, in the meantime, they received no intervention. Intervention subjects received a 6-month weight-loss intervention that is described in more detail elsewhere and was a programmatic adaptation of The 'I' Diet (SB Roberts and BK Sargent, http://www.theidiet.com). The overarching goal of the intervention was to help participants achieve a sustainable weight loss of 0.5–1.0 kg per week by participation in a group-delivered behavioral program designed to facilitate adherence to recommendations to reduce energy intake by 500–1000 k cal per day, with novel intervention components included to facilitate sustainability of reducing energy intake via reducing hunger and devaluing existing associations between unhealthy food consumption and reward in parallel with reinforcing associations between healthy food consumption and reward.

The intervention was delivered in-person to groups of 15–20 participants by nutrition professionals with experience in behavioral weight management programs. There was a total of nineteen 60-min didactic and support group sessions to deliver during the course of the 24-week intervention (15 weekly sessions followed by 4 biweekly sessions), 16 sessions were presented to the groups on average (some sessions were missed due to snow days and a holiday) and average attendance at presented sessions was 84%.^10^ In addition to the group sessions, participants received a weekly email from their nutritionist for individual support.

Specific behavior changes taught and supported in the intervention included the use of portion-controlled menus and recipe suggestions, with high-satiety menu plans, recipes and tip sheets provided by the investigators. The menu plans had a specific dietary composition profile that combined low-glycemic index carbohydrates with higher fiber and higher protein—that is, foods with a slower digestion profile and reduced fluctuations in blood glucose that, on theoretical grounds, could reduce hunger. In particular, they provided approximately 25 % energy from protein and fat, and 50 % from low-glycemic index carbohydrates and contained ⩾40 g  per day dietary fiber. These specific dietary targets are different from those of typical behavioral weight control interventions,^13^ which have flexible macronutrient ranges rather than specific targets. The protein target of this intervention was at the higher end of the Acceptable Macronutrient Distribution Ranges of the Dietary Reference Intakes,^14^ and the dietary fiber target was higher than national recommendations but similar to amounts tested previously for reduction in cardiometabolic risk factors.^15^ The low-glycemic index recommendation in the intervention is consistent with a recent Cochrane review of glycemic index, indicating a benefit of low-glycemic index menus for weight loss,^16^ whereas typical behavioral interventions do not currently recommend based on glycemic index.^13^ A variety of standard behavior change elements were included in the intervention to support generalized behavior changes for reducing energy intake including meal planning, goal setting and motivation.^17^ We also included additional topics specific to supporting adherence to the program's novel goals for dietary composition, hunger reduction and food cravings reduction. In addition to the provided menus, these included information on evenly spacing meals and snacks, and the use of ‘free foods' (specific listed foods with few calories that could be eaten *ad libitum*) for acute hunger relief.

### fMRI image acquisition

**Figure 1:**
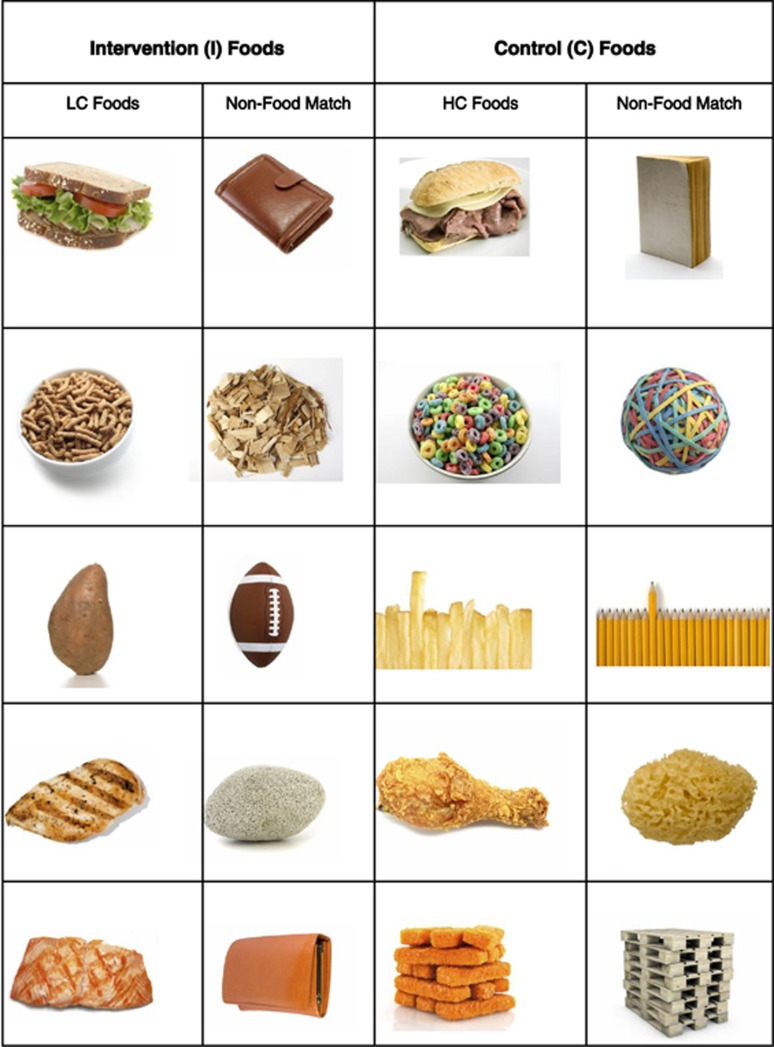
Examples of the high-calorie (HC) and low-calorie (LC) images used for the functional magnetic resonance imaging scans, together with the paired non-food images that were matched to each food picture for approximate color, size and image complexity. Information on nutrient composition differences between food groups are given in Table 2.

fMRI blood oxygen-level-dependent scans were conducted prior to randomization and at 6 months to assess neuronal activity in the striatum, a region strongly implicated in reward processing by previous work.^18,19^ Forty food and forty non-food (NF) images were used. The food images were grouped into two categories that, for simplicity, are called HC (*n*=20) and LC (*n*=20). HC food images in this study were of typical HC foods eaten regularly in the United States, which also usually have low-dietary fiber content and high-energy density and glycemic index. LC food images were of foods that were consistent with the specific dietary composition recommendations of the intervention, being relatively low in calories for the type of food and with higher fiber, low-glycemic index and/or high protein content. To the extent possible, HC and LC foods were matched between groups for their similar functions in daily eating (for example, equivalent number of breakfast foods, snacks and so on). Each food image was paired with a NF image that had roughly comparable color, size and visual complexity, and NF data were subtracted out from all analyses. Table 2 shows complete list of all the foods by HC and LC designation together with mean dietary composition values, and Figure 1 shows examples of the images.

Structural (MPRAGE) and functional (T2*-weighted) MRIs were acquired at the Athinoula A. Martinos Center for Biomedical Imaging of the Massachusetts General Hospital (Charlestown, MA, USA) using a Siemens 3.0-T Trio whole-body scanner (Erlangen, Germany). Image scanning took approximately 45 min per scanning session, and took place approximately 4 h after subjects had eaten a meal consistent with their randomized group,^20^ which is a time interval that approximates typical eating intervals for humans. During scanning, subjects viewed the 40 food and 40 NF images and rated desirability using a button box with a scale of 1–4, where 1 was ‘not desirable at all' and 4 was ‘extremely desirable'. Each trial (picture presentation) lasted for 5 s. There were 20 trials in each condition (HC images, HC-NF images, LC images, LC-NF images). Trials were presented in random order interspersed with fixation cross trials of varying lengths. All conditions were presented once across four runs. The four categories of pictures were counterbalanced across participants.

### Data analyses

Preprocessing and statistical analysis of the fMRI data were performed using SPM8 software (Wellcome Department of Cognitive Neurology, London, UK; http://www.fil.ion.ucl.ac.uk/spm/) and using the Matlab7.4 platform (Natick, MA, USA). Images were corrected for motion and normalized to the standard space established by the Montreal NeurologiHC Institute (www.bic.mni.mcgill.ca), resampled to 2 mm voxels and smoothed with a 3D Gaussian kernel of 6 mm width (full-width at half-maximum). A general linear model was applied to the time series convolved with the canonical hemodynamic response function and a 128 s high-pass filter. Images were adjusted for global confounds (for example, movement). Condition effects (HC-NF, LC-NF) were estimated on an individual level (first-level analysis) at each voxel, and statistical parametric maps (that is, con images) were created for each condition at baseline and 6 months representing the β-weight for each condition. These contrasts, or statistical parametric maps, were entered into a second-level random-effects analysis to estimate condition effects on the group level using a flexible factorial model. We examined the interaction term for HC versus LC foods (corrected for NF values), baseline versus 6-month scans and intervention group versus control group in *a priori* regions of interest. The regions of interest of the striatum (caudate and putamen) was defined using masks provided by the Anatomical Automatic Labeling in the WFU Pickatlas toolbox (Winston-Salem, NC, USA). We used a statistical threshold of *P* (uncorrectd) <0.05 and cluster width of at least 5 contiguous voxels. The statistical parametric maps toolbox MarsBar (MRHC Cognition and Brain Sciences Unite, Cambridge, UK) was used to engage the statistical analyses of the regions of interest.

Analyses of directional changes in blood oxygen-level-dependent signal activation values in response to HC and LC food images also used data with the corresponding NF activations subtracted out. Change in the relative responsiveness to HC and LC foods was calculated for both regions at baseline and 6 months as HC activation minus LC activation, and baseline values were subtracted from the 6-month values to calculate mean change over time. Within-group mean change values were compared with 0 using two-sided paired *t*-tests and between-group comparisons were made using two-sided independent *t*-tests. Cohen's *d*-effect size calculations were made to evaluate the size of the mean changes between-groups and within-groups for baseline to 6-month scans. Baseline, 6 months and change in subject characteristics and eating behavior measures were compared between the control and intervention subjects using two-sided independent *t*-tests and Fisher's exact test for the baseline differences in sex proportions. These statistical analyses were performed by using SAS version 9.3 (Cary, NC, USA) and IBM SPSS Statistics version 22 (Armonk, NY, USA) and significance was set at *α*=0.05. Data are presented as means and s.e.m.

## Results

**Figure 2:**
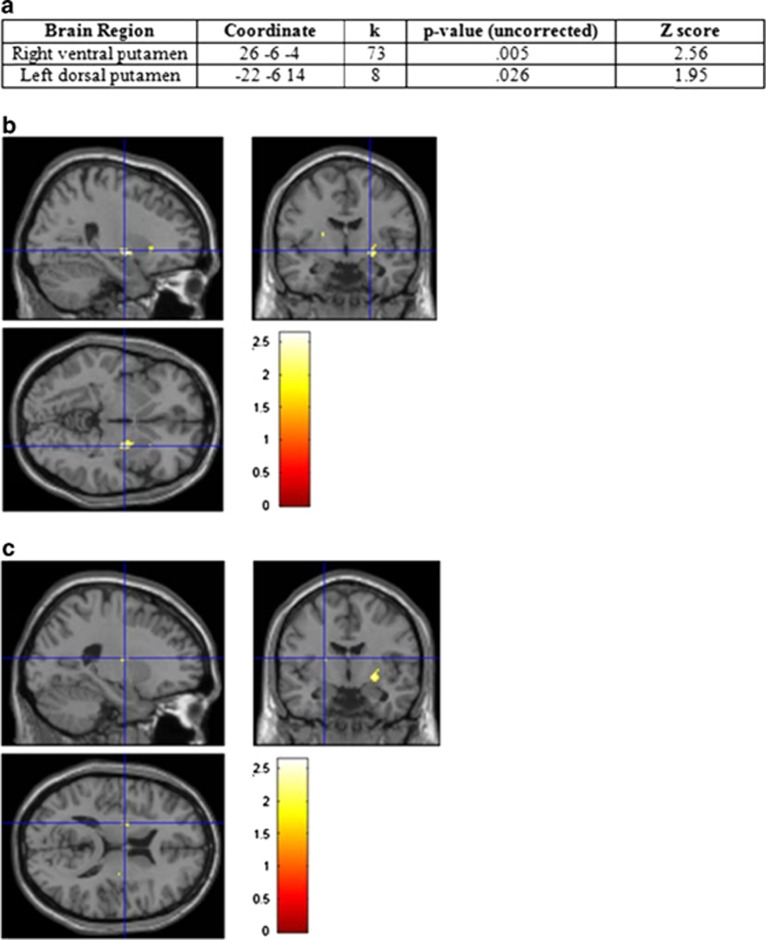
(**a**) Significant locit identified for differences between control and intervention participants in changes in functional magnetic resonance imaging blood oxygen-level-dependent activation from baseline to 6 months in response to viewing 20 pictures of high-calorie (HC) and 20 pictures of low-calorie (LC) foods, as well as a non-food (NF) control images matched to each food picture for approximate color, size and visual complexity. Data presented are with data for non-food images subtracted out. (**b**) The right ventral putamen at max voxel coordinate *x*=26, *y*=−6 and *z*=−4, *P*=0.005 with 73 contiguous voxels above threshold. (**c**) The left dorsal putamen at max voxel coordinate *x*=−22, *y*=−6 and *z*=14, *P*=0.026, eight contiguous voxels above threshold (**c**). Note, there was also a one-voxel above threshold activation in the right dorsal putamen *x*=30, *y*=−16 and *z*=12, *Z*-score=1.75, *P*=0.04.

Subjects randomized to the behavioral intervention achieved significant weight loss versus controls (−6.3±1.0 kg versus +2.1±1.1 kg, *P*<0.001). Two regions in the dorsal and ventral striatum were identified where there were significant differences between the groups in changes (6 months to baseline) in fMRI blood oxygen-level-dependent activation to the food images corrected for the paired NF images, which did not include areas such as the caudate that have previously been implicated as important in reward system responsiveness to food in humans. As illustrated in Figure 2, the areas with significant changes were the right ventral putamen (coordinate *x*=26, *y*=−6 and *z*=−4) and the left dorsal putamen (coordinate *x*=−22, *y*=−6 and *z*=14).

**Figure 3:**
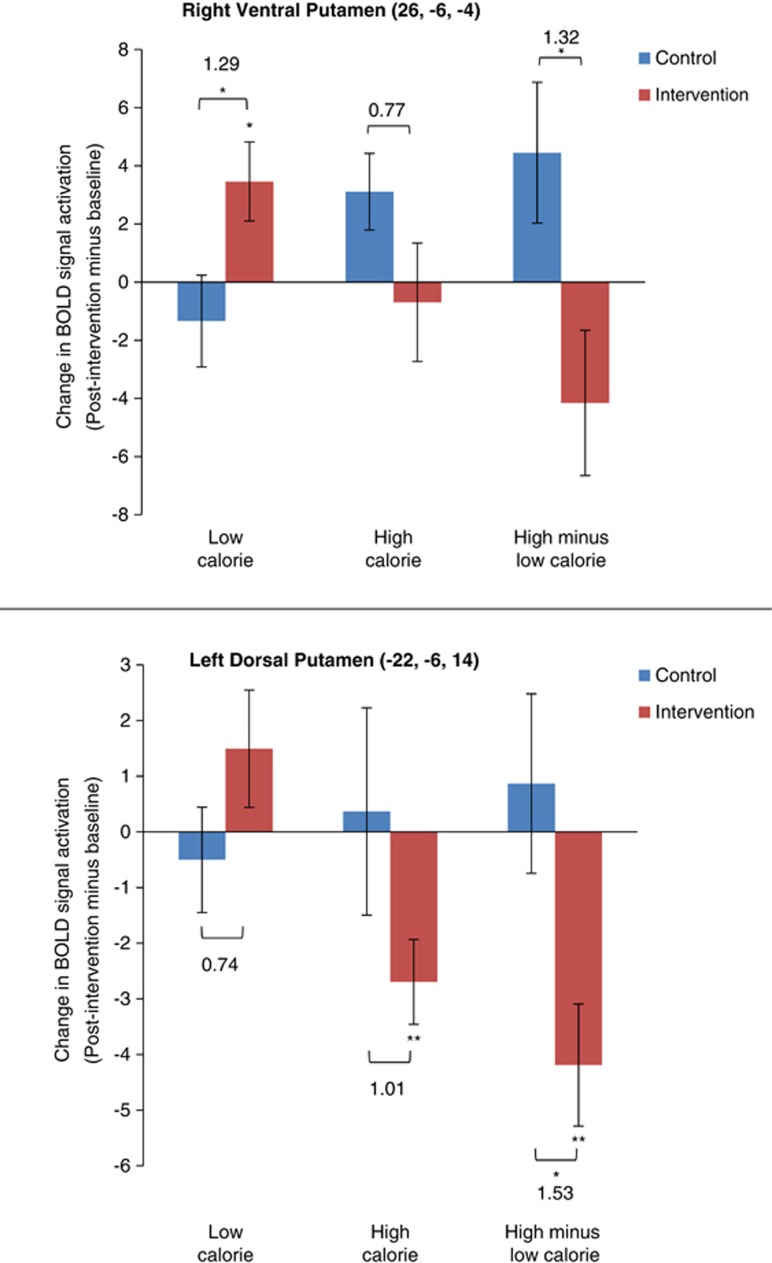
Differences between groups in changes over time (6 months to baseline) in blood oxygen-level-dependent (BOLD) signal *b*-values (max-voxels) for high-calorie (HC) and low-calorie (LC) foods minus non-food (NF) paired images; also, changes in relative signal strength for HC and LC (delta values for HC-NF minus LC-NF). Significance is denoted with *P<0.05 or **P<0.01, and relevant Cohen's *d*-values are given as numerals.

**Figure 4:**
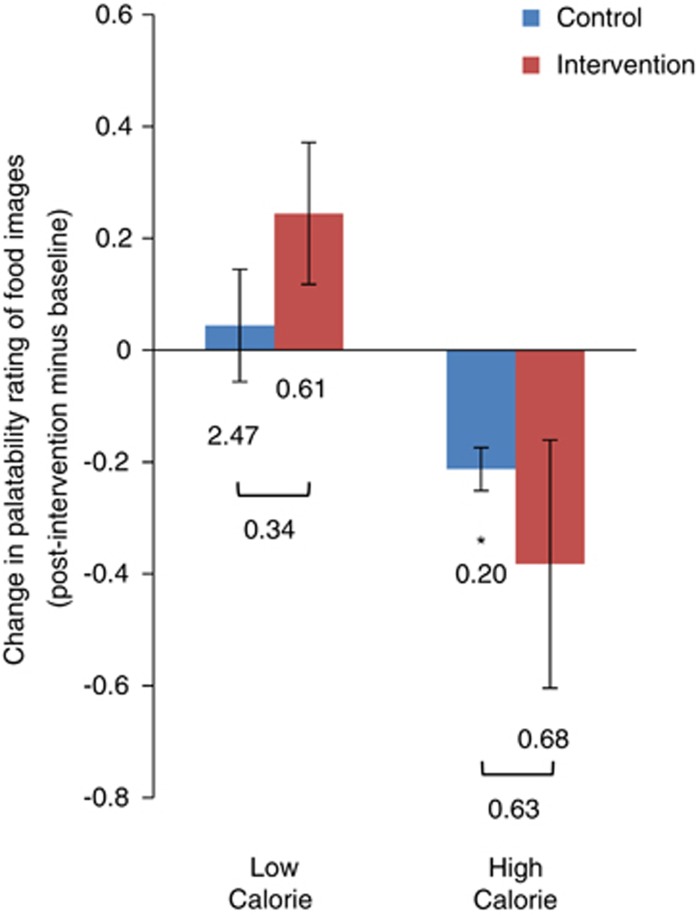
While in the scanner, subjects rated the desirability of the images on a scale of 1–4, with 1 being ‘not at all desirable' and 4 being ‘extremely desirable.' Data are means and s.e.m. Numbers are Cohen's *d*-values for paired and independent comparisons. The change in high-calorie food desirability was significantly different from 0 in the controls (*P<0.01).

We also examined the directional changes in blood oxygen-level-dependent responses to HC and LC food images over time. As shown in Figure 3, there were opposite changes over time in intervention versus control participants in activation for HC versus LC foods. Specifically, although there was no significant change in mean activation for all foods combined in the two groups (*P*=0.77, right ventral putamen; *P*=0.70, left dorsal putamen), mean activation increased significantly for LC foods in the right ventral putamen and decreased significantly for HC foods in the left dorsal putamen in intervention subjects versus controls. We also calculated the relative directional shift in responsiveness to HC versus LC foods in both regions from baseline to 6 months, as HC activation minus LC activation with baseline values subtracted from the 6-month values. These directional shifts were significantly different between groups and of large magnitude in both brain regions; specifically, there was an increase in the relative signal for HC versus LC foods in controls and a large negative change in the intervention participants. Moreover, the shift in the left dorsal putamen in the intervention participants was of large magnitude and was a significant absolute change from baseline. Thus, in contrast to control subjects, the balance of responsiveness to HC versus LC foods in both brain regions shifted in favor of greater relative activation for LC foods versus HC in intervention participants, a finding that is consistent with the mean changes in food preference ratings for the food images assessed during MRI scanning (Figure 4). Changes over time within the intervention group were not significantly correlated with eating behavior characteristics or weight change in this small sample (*n*=8).

## Discussion

To our knowledge, these results provide the first randomized controlled trial data for demonstration and localization of changes in reward system activity with a behavioral weight-loss program versus wait-listed control. Moreover, while the regulation of food intake via reward systems is clearly complicated,^21^ the fact that changes were identified in both the dorsal and the ventral striatum suggests that broad changes occurred in reward system responsiveness that potentially can impact the valuation of different foods both at the level of anticipation of consumption and at the level of actual consumption.^22^

Two previous nonrandomized studies examined changes in reward system activation in behavioral weight loss interventions and found no changes in reward system activation over time,^6,7^ while nonrandomized studies of gastric bypass and gastric banding have reported comparable reductions in reward system activation for HC foods,^23, 24^ findings that are consistent with studies of food reward in animal models of gastric bypass.^25^ In addition, one previous study of reward system activation following weight loss with a liquid calorie diet reported a substantial increase in reward system activation for a variety of foods.^26^ Thus, this study is, to our knowledge, the first demonstration of beneficial modifications in reward system responsiveness to different foods by behavioral intervention. It is also noteworthy that the observed changes in reward system responsiveness to food images in this study was associated with favorable behavior changes, as changes in food intake and/or physical activity must have occurred to effect the negative energy balance that was observed. Although this study cannot distinguish between the effects of weight loss and the effects of specific intervention components, as the first demonstration that favorable changes in neural responsivity can be achieved in a behavioral weight-loss program, it has important implications for obesity treatment.

This study also provides the first demonstration of significant changes in relative reward system activation for HC versus LC foods in a behavioral intervention and provides data consistent with the previous gastric bypass and banding observations.^23, 24^ However, in contrast to that study, which showed only a decrease in activation for HC foods, our intervention participants also had an absolute increase in activation for LC foods. Together these observations indicate that, compared to controls, participants in the intervention experienced a relative devaluation of anticipated reward for eating HC foods combined with amplified anticipated reward for eating LC foods, alterations that could potentially facilitate a desirable shift toward greater consumption of LC versus HC foods. In addition to the possibility that such changes may lead to greater sustainability of weight loss, which can be examined further in future studies, the results also address the generalized concern that maladaptive neuroplasticity in reward systems might not be reversible.^27, 28, 29^

It is important to note that this study involved a small number of subjects and thus trials with larger populations that can comprehensively investigate different brain areas involved in the regulation of food intake and include a long-term follow-up are now needed. Moreover, baseline dietary restraint was different between groups, which may possibly have influenced the results obtained, and a larger study population will also address concerns relating to matching all subject characteristics between groups. In addition, we studied overweight and Class I obese individuals, whereas morbidly obese individuals may have metabolic and/or genetic differences that makes them more resistant to treatment.^30,31^ It was also not possible to separate the effects of achieved weight loss from the specific effects of our intervention, so we do not yet know whether any of the novel intervention components designed to suppress hunger and food cravings contributed to the results obtained. However, it may be noteworthy that our intervention discouraged consumption of high-glycemic index carbohydrates, and high-glycemic index carbohydrates enhance reward center activation,^32^ suggesting that further exploration of this dietary profile is warranted.

In conclusion, based on the findings of this study, interventions that harness the potential for neuroplasticity in reward system responsiveness to HC versus LC food cues appear to be possible and can be explored further for their potential to enhance the effectiveness and sustainability of behavioral treatment of obesity.

## Figures and Tables

**Table 1:** Characteristics and eating behavior scores at baseline and 6 months in intervention and control participants

	*Control*		*Intervention*		P-*values*
	N	*Mean (s.e.m.)*	N	*Mean (s.e.m.)*	
Baseline					
Sex (% female)	5	4 (80)	8	4 (50)	0.56
Age	5	53.40 (5.10)	8	47.38 (3.93)	0.37
Height (m)	5	1.65 (0.03)	8	1.70 (0.04)	0.40
Weight (kg)	5	82.94 (4.88)	8	83.18 (2.77)	0.96
Body mass index	5	30.42 (1.75)	8	28.87 (0.82)	0.38
Craving inventory—trait score	4	138.00 (17.93)	5	88.20 (23.35)	0.15
Hunger score	4	8.25 (1.65)	6	2.67 (0.76)	0.01
Disinhibition score	4	12.50 (0.96)	6	6.33 (2.14)	0.05
Restraint score	4	6.58 (2.06)	6	6.67 (1.41)	0.97
					
6 Months					
Weight (kg)	5	85.07 (5.59)	8	76.89 (3.17)	0.19
Body mass index	5	31.21 (2.07)	8	26.71 (1.09)	0.06
Craving inventory—trait score	4	151.58 (13.70)	5	67.63 (12.96)	<0.01
Hunger score	4	10.25 (1.11)	6	2.96 (1.66)	0.01
Disinhibition score	4	13.97 (0.71)	6	5.15 (1.33)	0.001
Restraint score	4	5.83 (2.10)	6	15.06 (0.65)	0.001
					
Change (6 months minus baseline)					
Weight (kg)	5	2.14 (1.14)	8	−6.30 (1.04)	<0.001
Body mass index	5	0.79 (0.44)	8	−2.16 (0.32)	<0.001
Craving inventory—trait score	4	13.58 (22.11)	6	−20.57 (11.33)	0.19
Hunger score	4	2.00 (0.71)	7	0.29 (1.42)	0.39
Disinhibition score	4	1.47 (0.67)	7	−1.18 (1.96)	0.32
Restraint score	4	−0.75 (2.02)	7	8.39 (1.21)	<0.01

Subjects in this functional magnetic resonance imaging (fMRI) study were drawn from a larger randomized trial of the intervention versus a wait-listed control group. Not all participants in the fMRI studies completed the questionnaires and numbers shown here are for completers at both time points. In the larger population, changes in all variables noted here were significant.^8, 9, 10^ Differences between groups in changes over time in weight and body mass index were analyzed in the log 10 scale to approximate the normal distribution. Craving trait and dietary restraint, hunger and disinhibition were measured assessed as described in Subjects and Methods section.

**Table 2:** Food images used for the functional magnetic resonance imaging scans

*Low-calorie food images*	
Turkey sandwich on whole-wheat bread w/ lettuce & tomato	
Bowl fiber cereal	
Apple	
Baked sweet potato	
Frozen yogurt with berries	
Green salad with tomatoes	
Bean or lentil soup	
Grilled chicken	
Pasta with meat sauce	
Dark chocolate	
Walnuts	
Granola bar	
Sliced raw vegetables with hummus	
Diet Coke	
Baked salmon	
Whole-wheat pita pizza	
Peanut butter toast	
Bran muffin	
Egg-white omelet with vegetables	
Cup of coffee	

*Mean nutritional contents per 100 g*	
Energy (kcal)	205
Protein (g)	9.0
Fat (g)	9.3
Total carbohydrate (g)	26.9
Fiber (g)	10.3


*High-calorie food images*	
Roast beef and cheese sandwich on white bread w/o vegetables	
Bowl fruit loops	
Canned fruit	
French fries	
Ice cream with chocolate sauce	
Potato salad	
Cream-based soup	
Fried chicken	
Macaroni and cheese	
Truffles	
Chocolate turtle	
Chocolate chip cookie	
Chips	
Regular coke	
Fish sticks	
Personal pizza	
Buttermilk pancakes with maple syrup	
Donut	
Fried egg	
Frappuccino	
	
*Mean nutritional contents per 100 g*	
Energy (kcal)	284
Protein (g)	6.3
Fat (g)	14.7
Total carbohydrate (g)	32.9
Fiber (g)	2.4

## Notes

SBR is co-founder of the iDiet program (http://www.theidiet.com), which is a commercial weight loss program based on the intervention described in this paper. The remaining authors declare no conflict of interest.
